# Socioeconomic patient characteristics predict delay in cancer diagnosis: a Danish cohort study

**DOI:** 10.1186/1472-6963-8-49

**Published:** 2008-02-28

**Authors:** Rikke Pilegaard Hansen, Frede Olesen, Henrik Toft Sørensen, Ineta Sokolowski, Jens Søndergaard

**Affiliations:** 1 Research Unit and Department of General Practice, University of Aarhus, Vennelyst Boulevard 6, DK-8000 Aarhus C, Denmark; 2 Research Unit for General Practice, University of Aarhus, Vennelyst Boulevard 6, DK-8000 Aarhus C, Denmark; 3 Department of Clinical Epidemiology, Aarhus University Hospital, Ole Worms Allé 150, DK-8000 Aarhus C, Denmark; 4 Department of Epidemiology, Boston University School of Public Health, Boston, Massachusetts, USA

## Abstract

**Background:**

Delay in cancer diagnosis may be important for cancer prognosis. Large individual variations in the duration of delay have been observed. This study examines whether patients' socioeconomic characteristics are predictors of long patient-, doctor- and system-related delay in cancer diagnosis.

**Methods:**

Danish population-based cohort study. From September 2004 to September 2005, newly diagnosed cancer patients were enrolled from administrative registries. A total of 467 general practitioners in the County of Aarhus, Denmark, completed questionnaires on 2,212 cancer patients' diagnostic pathways. A total of 1,252 cancer patients filled in questionnaires on their socioeconomic characteristics (e.g. marital status, education, occupation, household income and fortune). Delay was categorised as short or long based on quartiles. Predictors of long delay were assessed in a logistic regression model using odds ratios (ORs) as a proxy of relative risks.

**Results:**

In regard to *patient delay*, retired female patients experienced shorter delays (OR 0.35, 95% confidence interval (95%CI) 0.13 to 0.98) than employed female patients, while female smokers experienced longer delays (OR 2.42, 95%CI 1.21 to 4.85) than female non-smokers.

In regard to *doctor delay*, female patients with a large household fortune experienced shorter delays (OR 0.07, 95%CI 0.01 to 0.45) than economically less privileged female patients. Well-educated men experienced shorter delays (OR 0.40, 95%CI 0.16 to 1.00) than men with short education. Male patients experienced longer doctor delays (OR 2.11, 95%CI 1.11 to 4.02) than women when gender-specific cancers were excluded.

In regard to *system delay*, female patients with a large household fortune experienced shorter delays (OR 0.46, 95%CI 0.21 to 0.99) than economically less privileged women, while female patients with a high alcohol intake experienced longer delays (OR 2.82, 95%CI 1.18 to 6.72) than women with an average intake.

**Conclusion:**

We found socioeconomic predictors of delay that allow us to hypothesize social inequalities in the distribution of delay, but, in general, only a few socioeconomic variables predicted delay in cancer diagnosis. Future research should examine a broader array of patients' personal characteristics.

## Background

Delay in cancer diagnosis and treatment may be an important factor for prognosis [1-6]. Delay also has deleterious psychological consequences for patients awaiting clarification of their disease [7]. Recent years have devoted growing attention to how different factors affect delay in cancer diagnosis as reducing delay may increase the proportion of early stage cancers and thereby result in improved survival. Coping strategies and help-seeking behaviour seem to be related to personal and socioeconomic patient characteristics [8-12]. For instance, it has been hypothesized that men are more reluctant than women to consult their general practitioners (GPs) when they experience potentially cancer-related symptoms [7,13,14]. This may reflect gender differences in the way patients cope with symptoms. In addition, patients' interaction with the GPs and the secondary health care sector may depend on their socioeconomic characteristics, which may thus play an important role for their help-seeking behaviour. If such associations do exist between socioeconomic patient characteristics and delay, this should influence the design of tailored, targeted interventions aimed at reducing delay (e.g. campaigns targeted at specific age groups, social classes etc.).

Previous research findings on potential socioeconomic characteristics associated with delay have been inconsistent and research has tended to focus on a few specific cancers. Neal and Allgar [9] found that long patient delay was associated with old age (lung cancer and non-Hodgkin's lymphoma) and being single (colorectal and breast cancer), and that long doctor delay was associated with old age (colorectal, lung, prostate, non-Hodgkin's lymphoma and breast cancer) and being single (breast cancer). Furthermore, old age (colorectal, prostate, non-Hodgkin's lymphoma and breast cancer), being single (colorectal, prostate, non-Hodgkin's lymphoma and breast cancer) and low social class (colorectal, ovarian, prostate and breast cancer) were associated with long system delay. Risberg et al [7] found no associations between patient delay and age, gender or education, but an association was found between short doctor delay and patients being well-educated and young in a study population of mixed cancers. Ramirez et al [10] concluded in a review of breast cancer patients that there was moderate or strong evidence for associations between long patient delay and old age and low education, while this type of delay was unrelated to marital and socioeconomic status. Furthermore, young age predicted long doctor delay. Montella et al [15] found associations between long patient delay and old age and low education while Burgess et al [16] found no association between patient delay and age, marital and socioeconomic status for breast cancer patients. Given the inconsistent findings in the literature our aim was to clarify whether specific socioeconomic patient characteristics predict long delay in the diagnosis of cancer in general.

## Methods

### Study design

We conducted a cohort study set in the County of Aarhus, Denmark with 640,000 inhabitants and approximately 3,000 new cancer cases diagnosed per year. Denmark's publicly funded health care system provides free access to general practice and hospital care. More than 98% of Danish citizens are registered with a GP [17,18], who functions as a gatekeeper to the remaining health care system, carrying out initial diagnostic investigations and referring patients to hospitals or outpatient clinics as needed. Danish GPs are required to keep detailed electronic medical records.

Our study population included all newly diagnosed cancer patients in Aarhus County during the 1-year period from 1 September 2004 to 31 August 2005. Patients younger than 18 years and those with non-melanoma skin cancers were excluded.

Patients were identified from the County Hospital Discharge Registry (HDR) that, for each hospital admission and outpatient visit, records the patient's unique civil registration number (CRN) [19], dates of admission and discharge and discharge diagnoses classified according to the International Classification of Diseases (ICD-10). We included all patients with at least one cancer diagnosis documented in the HDR during the study period, except those with a cancer recurrence. We then linked the HDR data to the County Health Service Registry (HSR) to identify each patient's GP.

### Data collection

Our data sources were data from a GP questionnaire on each patient's diagnostic pathway and data on socioeconomic characteristics obtained from a patient questionnaire.

A questionnaire was sent to the GP of each identified patient. The questionnaire asked for confirmation of the diagnosis and a detailed description of the patient's diagnostic pathway extracted from the electronic medical record and discharge letters from hospitals and specialists (e.g. dates of reported symptoms, encounters, tests, referrals and involvement of other providers). In practices with more than one GP, we asked the GP most familiar with the patient to complete the questionnaire. The GPs received compensation for their participation. We also sent a questionnaire to patients as soon as they were identified in the HDR. This questionnaire requested information on specific socioeconomic characteristics selected on the basis of critical literature studies, such as marital status, number of children, education, occupation, household income and fortune, smoking and alcohol habits. The questions were adapted and modified from the population survey questionnaire from the Danish Institute of Public Health [20]. Non-responders received a reminder after three weeks.

### Outcome measures

**Figure 1:**
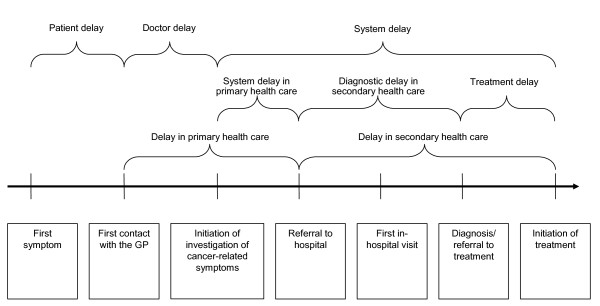
Catagorisation of delay.

Delay was calculated on the basis of dates provided by the GPs. As shown in Figure 1, three sources of delay were identified: *patient delay *(median 21 days, interquartile interval (IQI) 7 to 56), *doctor delay *(median 0, IQI 0 to 2) and *system delay *(median 55, IQI 32 to 93) (Hansen et al: Where does delay occur in cancer diagnosis? A cohort study of delay duration in 2,212 newly diagnosed cancer patients, submitted). Delay was categorised as either short or long delay, with long delay defined as the 4^th ^quartile of all patients' delay and the remaining delay defined as short. Because the 75^th ^percentile for doctor delay was only 2 days (see above), we used a cut-off of 30 days (corresponding to the 91^st ^percentile) to classify short versus long doctor delay. It is clinically appropriate for patients and GPs, as watchful waiting of a few weeks' duration is often a part of a standard diagnostic investigation [21]. Thus, long patient delay was set to > 60 days, long doctor delay to > 30 days and long system delay to > 90 days.

### Analyses

The analyses were restricted to pathways in which a GP was involved in the diagnosis. Other pathways could be emergency or out-of-hours cases, which were excluded.

We used logistic regression analyses to quantify whether socioeconomic patient characteristics predicted long delay. We included all covariates in multivariate analyses after having assessed for collinearity. The variables included were age, marital status, having children, education, occupation, household income and fortune, smoking and alcohol intake. We accounted for the clustering of patients within GPs by using robust variance estimates [22] in both univariate and multivariate models. The estimates are presented as odds ratios (ORs) as a proxy of the relative risks with 95% confidence intervals (95%CIs). Additional analyses were performed after exclusion of gender-specific cancers (breast cancer and female/male genital cancers). Data were analysed using Stata 9.2.

### Ethics approval

According to the Scientific Committee for the County of Aarhus, the Biomedical Research Ethics Committee System Act does not apply to this project. The study was approved by the Danish Data Protection Agency and the Danish National Board of Health.

## Results

**Figure 2:**
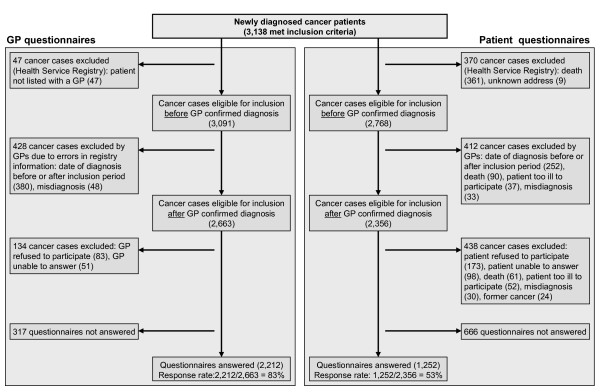
Flowchart.

Among a total of 543 GPs, 467 (86%) from 255 general practices filled in 2,212 questionnaires. On average, the GPs completed questionnaires for 4.7 patients (interval 1–15, median 4). General practices were involved in the diagnostic investigation in 1,892 (86%) of the 2,212 cancer cases. Among a total of 2,356 patients, 1,252 (53%) completed a questionnaire. Figure 2 shows inclusion, exclusion and non-response characteristics for physicians and patients. Analyses of non-responders revealed no major discrepancies between participating and non-participating patients and GPs with respect to age, gender or distribution of cancer diagnoses (data not shown). Table 1 shows the distribution of the different cancer types in the study.

**Table 1:** Cancers in the study. The number (N) of different cancer types in the study.

**Cancer type**	**N**
All Cancers	1892
Breast cancer	291
Colorectal cancer	254
Lung cancer	253
Prostate cancer	190
Melanoma	122
Bladder cancer	73
Non-Hodgkin's lymphoma	54
Pancreas cancer	54
Ovarian cancer	47
Corpus uteri cancer	41
Other	513

### Gender and delay

We found that male cancer patients experienced longer *doctor delays *(OR 1.65, 95%CI 1.19 to 2.28) and *system delays *(OR 1.86, 95%CI 1.48 to 2.35), but not longer *patient delays *(OR 1.28, 95%CI 0.95 to 1.71) than female cancer patients (Table 2). However, gender was not associated with long delay in univariate analyses when gender-specific cancers were excluded.

**Table 2:** Univariate analyses for gender and delay. Univariate analyses for gender and delay for all cancers and after exclusion of gender-specific cancers, accounting for patient clustering within general practitioners. N in each column is the number of answers with complete data with the number of patients with long delays provided in brackets. Results are presented as odds ratios (ORs) with 95% confidence intervals (95%CIs).

		**Patient delay; OR(95%CI)**		**Doctor delay; OR(95%CI)**		**System delay; OR(95%CI)**	
		**N**	**Univariate**	**N**	**Univariate**	**N**	**Univariate**

All	Female	634(135)	1	994(70)	1	813(172)	1
	Male	603(155)	1.28(0.95 to 1.71)	883(98)	**1.65(1.19 to 2.28)**	609(203)	**1.86(1.48 to 2.35)**
Without gender	Female	373(81)	1	575(53)	1	428(119)	1
specific cancer	Male	471(107)	1.06(0.74 to 1.52)	670(77)	1.28(0.87 to 1.88)	469(136)	1.06(0.81 to 1.39)

### Socioeconomic patient characteristics and delay

Stratified analyses of socioeconomic patient characteristics and delay are shown in Table 3 for women and Table 4 for men. Only results from the multivariate analyses are presented in the text. The parameters in the multivariate analyses for the different delay types explained 6–12% of the variance for women and 3–6% for men. We conducted Hosmer-Lemeshow goodness-of-fit tests on our multivariate models and found that the models fitted to a satisfactory extent. We controlled for interactions between the different parameters and found no relevant significant associations.

**Table 3:** Analyses for socioeconomic patient characteristics and delay (women). Univariate and multivariate analyses for socioeconomic patient characteristics and the three delay stages in *women*, accounting for patient clustering within general practitioners. N in each column is the number of answers with complete data with the number of patients with long delays provided in brackets. Results are presented as odds ratios (ORs) with 95% confidence intervals (95%CIs).

**Predictors**		**Patient delay; OR(95%CI)**				**Doctor delay; OR(95%CI)**				**System delay; OR(95%CI)**			
		**N**	**Univariate**	**N**	**Multivariate**	**N**	**Univariate**	**N**	**Multivariate**	**N**	**Univariate**	**N**	**Multivariate**

Age	18–49	102(23)	0.91(0.51 to 1.63)	62(17)	0.63(0.29 to 1.39	177(13)	1.31(0.65 to 2.64	113(9)	0.92(0.35 to 2.39	162(35)	1.05(0.66 to 1.67	105(20)	0.78(0.39 to 1.53
	50–69	265(64)	1	136(41)	1	403(23)	1	212(14)	1	351(73)	1	198(35)	1
	70+	267(48)	0.69(0.44 to 1.08)	70(8)	0.72(0.25 to 2.09)	414(34)	1.48(0.87 to 2.50)	104(8)	1.85(0.42 to 8.21)	300(64)	1.03(0.70 to 1.53)	88(16)	1.26(0.50 to 3.15)
Marital status	Cohabiting	197(53)	1	176(49)	1	322(20)	1	288(19)	1	301(48)	1	269(42)	1
	Single	114(24)	0.72(0.43 to 1.23)	92(17)	0.82(0.38 to 1.75)	180(15)	1.37(0.69 to 2.71)	141(12)	1.07(0.46 to 2.50)	156(35)	1.52(0.93 to 2.51)	122(29)	1.44(0.79 to 2.63)
Having Children	Yes	273(65)	1	235(55)	1	442(30)	1	375(27)	1	401(70)	1	341(59)	1
	No	36(11)	1.41(0.68 to 2.94)	33(11)	1.77(0.80 to 3.92)	59(5)	1.27(0.48 to 3.40)	54(4)	0.77(0.25 to 2.36)	55(13)	1.46(0.70 to 3.06)	50(12)	1.20(0.55 to 2.60)
Education	< 3 years	147(35)	1	134(33)	1	235(16)	1	209(15)	1	224(35)	1	199(28)	1
	≥ 3 years	83(22)	1.15(0.62 to 2.15	79(2)	1.12(0.55 to 2.27	143(5)	0.50(0.18 to 1.40	133(5)	0.45(0.15 to 1.35	128(26)	1.38(0.78 to 2.43)	118(26)	1.76(0.94 to 3.28
	No	72(18)	1.07(0.54 to 2.09)	55(12)	1.42(0.60 to 3.36)	110(13)	1.83(0.85 to 3.94)	87(11)	1.83(0.79 to 4.24)	96(21)	1.51(0.82 to 2.79)	74(17)	1.50(0.74 to 3.03)
Occupation	Working^1^	137(44)	1	129(42)	1	223(14)	1	210(14)	1	212(44)	1	199(40)	1
	Not working^2^	42(11)	0.75(0.34 to 1.67	32(10)	0.81(0.34 to 1.95	71(9)	2.17(0.89 to 5.29	54(8)	1.93(0.68 to 5.44	62(12)	0.92(0.47 to 1.79	46(8)	0.69(0.32 to 1.49
	Retirees^3^	130(23)	**0.45(0.26 to 0.81)**	107(14)	**0.35(0.13 to 0.98)**	206(12)	0.92(0.43 to 1.99)	165(9)	0.35(0.06 to 1.93)	181(27)	0.67(0.39 to 1.15)	146(23)	0.42(0.16 to 1.09)
Household income	Low/middle	221(51)	1	201(44)	1	362(25)	1	327(24)	1	324(62)	1	294(59)	1
	High	68(22)	1.59(0.88 to 2.89)	67(22)	1.30(0.60 to 2.82)	104(7)	0.97(0.41 to 2.28)	102(7)	1.50(0.50 to 4.53)	99(13)	0.64(0.34 to 1.21)	97(12)	0.55(0.26 to 1.19)
Household fortune	Small/medium	196(46)	1	185(42)	1	331(31)	1	313(30)	1	302(62)	1	287(60)	1
	Large	86(24)	1.26(0.73 to 2.18)	83(24)	1.36(0.72 to 2.56)	120(1)	**0.08(0.01 to 0.61)**	116(1)	**0.07(0.01 to 0.45)**	108(13)	0.53(0.27 to 1.02)	104(11)	**0.46(0.21 to 0.99)**
Smoking	No	240(53)	1	209(45)	1	378(27)	1	329(24)	1	343(61)	1	298(53)	1
	Yes	70(25)	**1.96(1.09 to 3.51)**	59(21)	**2.42(1.21 to 4.85)**	120(8)	0.93(0.41 to 2.12)	100(7)	0.57(0.23 to 1.41)	111(22)	1.14(0.65 to 2.00)	93(18)	1.01(0.56 to 1.84)
Alcohol intake per week^4^	≤ 14	287(73)	1	251(63)	1	464(31)	1	405(28)	1	424(75)	1	369(64)	1
	>14	17(3)	0.63(0.18 to 2.23)	17(3)	0.51(0.14 to 1.92)	26(3)	1.82(0.51 to 6.55)	24(3)	2.88(0.67 to 12.4)	23(7)	2.04(0.82 to 5.08)	22(7)	**2.82(1.18 to 6.72)**

^1^Employed patients, students

^2^Unemployed patients, patients on disability retirement, patients on personal leave or sick leave not caused by the cancer, housewives

^3^Early retired (age: 60–64) or retired (age: ≥ 65) employee

^4^According to recommendations from the Danish National Board of Health: maximum 21 units per week for men and 14 units per week for women

**Table 4:** Analyses for socioeconomic patient characteristics and delay (men). Univariate and multivariate analyses for socioeconomic patient characteristics and the three delay stages in *men*, accounting for patient clustering within general practitioners. N in each column is the number of answers with complete data with the number of patients with long delays provided in brackets. Results are presented as odds ratios (ORs) with 95% confidence intervals (95%CIs).

**Predictors**		**Patient delay; OR(95%CI)**				**Doctor delay; OR(95%CI)**				**System delay; OR(95%CI)**			
		**N**	**Univariate**	**N**	**Multivariate**	**N**	**Univariate**	N	**Multivariate**	**N**	**Univariate**	**N**	**Multivariate**

Age	18–49	52(9)	**0.43(0.20 to 0.92)**	25(5)	0.63(0.19 to 2.11)	78(8)	1.10(0.50 to 2.44)	39(3)	0.80(0.19 to 3.42)	68(13)	**0.53(0.28 to 0.99)**	37(8)	0.68(0.27 to 1.72)
	50–69	259(85)	1	109(37)	1	384(36)	1	167(21)	1	278(86)	1	134(43)	1
	70+	292(61)	**0.54(0.37 to 0.79)**	97(20)	0.45(0.20 to 1.00)	421(54)	1.42(0.96 to 2.10)	132(43)	1.07(0.47 to 2.45)	263(104)	**1.46(1.04 to 2.06)**	103(39)	1.25(0.57 to 2.76)
Marital status	Cohabiting	212(56)	1	185(49)	1	308(40)	1	270(37)	1	249(86)	1	220(72)	1
	Single	61(16)	0.99(0.50 to 1.95)	46(13)	1.18(0.46 to 3.06)	85(8)	0.70(0.32 to 1.51)	68(6)	0.45(0.17 to 1.18)	67(22)	0.93(0.50 to 1.71)	54(18)	0.96(0.44 to 2.10)
Having children	Yes	244(63)	1	208(54)	1	354(41)	1	308(39)	1	284(99)	1	250(84)	1
	No	24(8)	1.44(0.58 to 3.55)	23(8)	1.58(0.50 to 5.02)	32(5)	1.41(0.53 to 3.77)	30(4)	1.50(0.43 to 5.21)	26(7)	0.69(0.28 to 1.71)	24(6)	0.64(0.19 to 2.14)
Education	< 3 years	171(45)	1	143(37)	1	242(35)	1	208(32)	1	194(67)	1	168(55)	1
	≥ 3 years	77(23)	1.19(0.66 to 2.15	74(22)	1.02(0.49 to 2.13	110(6)	**0.34(0.14 to 0.81**	106(6)	**0.40(0.16 to 1.00**	90(29)	0.90(0.51 to 1.58)	86(27)	1.02(0.54 to 1.93
	No	17(3)	0.60(0.17 to 2.10)	14(3)	0.81(0.21 to 3.14)	31(6)	1.42(0.56 to 3.57)	24(5)	1.31(0.48 to 3.57)	25(9)	1.07(0.44 to 2.56)	20(8)	1.30(0.47 to 3.56)
Occupation	Working^1^	89(23)	1	82(21)	1	133(13)	1	125(11)	1	114(30)	1	108(27)	1
	Not working^2^	25(9)	1.61(0.66 to 3.92	21(9)	2.36(0.86 to 6.52	37(6)	1.79(0.62 to 5.17	31(6)	1.88(0.61 to 5.83	31(12)	1.77(0.73 to 4.28	27(12)	2.32(0.85 to 6.32
	Retirees^3^	158(39)	0.94(0.52 to 1.71)	128(32)	1.67(0.68 to 4.10)	222(29)	1.39(0.71 to 2.69)	182(26)	1.27(0.48 to 3.39)	170(64)	**1.69(1.02 to 2.79)**	139(51)	1.31(0.54 to 3.20)
Household income	Low/middle	207(52)	1	179(44)	1	293(42)	1	258(38)	1	233(82)	1	206(72)	1
	High	54(18)	1.49(0.75 to 2.95)	52(18)	1.23(0.46 to 3.30)	82(5)	0.39(0.15 to 1.04)	80(5)	0.59(0.20 to 1.75)	69(19)	0.70(0.38 to 1.29)	68(18)	1.02(0.44 to 2.37)
Household fortune	Small/medium	131(29)	1	116(25)	1	199(26)	1	183(26)	1	163(60)	1	151(53)	1
	Large	120(37)	1.57(0.88 to 2.80)	115(37)	1.83(0.89 to 3.78)	161(18)	0.84(0.44 to 1.60)	155(17)	0.86(0.41 to 1.80)	128(38)	0.72(0.44 to 1.20)	123(37)	0.77(0.42 to 1.41)
Smoking	No	204(52)	1	168(45)	1	296(36)	1	250(32)	1	241(80)	1	205(65)	1
	Yes	70(20)	1.17(0.64 to 2.15)	63(17)	1.03(0.50 to 2.13)	98(12)	1.01(0.50 to 2.02)	88(11)	0.86(0.42 to 1.79)	76(28)	1.17(0.71 to 1.95)	69(25)	1.11(0.61 to 2.01)
Alcohol intake per week^4^	≤21	237(58)	1	251(63)	1	345(42)	1	296(38)	1	275(92)	1	239(77)	1
	>21	31(13)	**2.23(1.05 to 4.73)**	17(3)	1.86(0.81 to 4.28)	43(5)	0.95(0.35 to 2.60)	42(5)	1.25(0.42 to 3.73)	36(13)	1.12(0.54 to 2.36)	35(13)	1.24(0.54 to 2.83)

^1^Working patients, students

^2^Unemployed patients, patients on disability retirement, patients on personal leave or sick leaves not caused by the cancer, housewives

^3^Early retired (age: 60–64) or retired (age: ≥ 65) employee

^4^According to recommendations from the Danish National Board of Health: maximum 21 units per week for men and 14 units per week for women

### Patient delay

Retired female cancer patients experienced shorter *patient delays *(OR 0.35, 95%CI 0.13 to 0.98) than employed female patients and smoking female patients experienced longer patient delays (OR 2.42, 95%CI 1.21 to 4.85) than non-smoking women. None of the characteristics under study predicted patient delay in men.

### Doctor delay

Female patients with a large household fortune experienced shorter *doctor delays *(OR 0.07, 95%CI 0.01 to 0.45) than economically less privileged female patients. Well-educated male patients experienced shorter doctor delays (OR 0.40, 95%CI 0.16 to 1.00) than men with short education.

### System delay

Female patients with a large household fortune experienced shorter *system delays *(OR 0.46, 95%CI 0.21 to 0.99) than economically less privileged female patients, while female patients with a high alcohol intake experienced longer system delays (OR 2.82, 95%CI 1.18 to 6.72) than women with an average intake. None of the characteristics under study predicted system delay in men.

### Exclusion of gender-specific cancers

Table 5 indicates that exclusion of patients with gender-specific cancers from the analyses did not significantly alter the estimates.

**Table 5:** Analyses for socioeconomic patient characteristics and delay (after exclusion of gender-specific cancers). Univariate and multivariate analyses for socioeconomic patient characteristics and the three delay stages in the patient population *after exclusion of gender-specific cancers*, accounting for patient clustering within general practitioners. N in each column is the number of answers with complete data with the number of patients with long delays provided in brackets. Results are presented as odds ratios (ORs) with 95% confidence intervals (95%CIs).

**Predictors**		**Patient delay; OR(95%CI)**				**Doctor delay; OR(95%CI)**				**System delay; OR(95%CI)**			
		**N**	**Univariate**	**N**	**Multivariate**	**N**	**Univariate**	**N**	**Multivariate**	**N**	**Univariate**	**N**	**Multivariate**

Gender	Female	373(81)	1	124(35)	1	575(53)	1	199(18)	1	428(119)	1	173(44)	1
	Male	471(107)	1.06(0.74 to 1.52)	164(42)	0.77(0.42 to 1.44)	670(77)	1.28(0.87 to 1.88)	233(34)	**2.11(1.11 to 4.02)**	469(136)	1.06(0.81 to 1.39)	200(54)	1.18(0.73 to 1.93)
Age	18–49	71(19)	0.94(0.52 to 1.71)	36(11)	1.00(0.41 to 2.46)	131(14)	1.19(0.65 to 2.17)	72(7)	0.95(0.37 to 2.42)	116(31)	1.00(0.64 to	66(16)	1.13(0.55 to 2.30)
	50–69	369(103)	1	152(53)	1	526(48)	1	221(27)	1	407(109)	1	192(48)	1
	70+	404(66)	0.50(0.36 to 0.71)	100(13)	**0.30(0.13 to 0.71)**	588(68)	1.30(0.91 to 1.86)	139(18)	1.34(0.51 to 3.54)	374(115)	1.21(0.89 to 1.66)	115(34)	1.15(0.50 to 2.63)
Marital status	Cohabiting	229(62)	1	204(58)	1	348(42)	1	310(39)	1	304(75)	1	273(66)	1
	Single	103(23)	0.77(0.45 to 1.35)	84(19)	0.98(0.51 to 1.90)	152(17)	0.92(0.51 to 1.64)	122(13)	0.67(0.33 to 1.36)	124(38)	1.35(0.85 to 2.14)	100(32)	1.07(0.60 to 1.89)
Having children	Yes	301(74)	1	262(67)	1	450(50)	1	390(46)	1	381(99)	1	334(86)	1
	No	28(10)	1.70(0.75 to 3.86)	26(10)	1.63(0.67 to 3.96)	46(8)	1.68(0.75 to 3.78)	42(6)	1.37(0.52 to 3.59)	43(13)	1.23(0.61 to 2.48)	39(12)	1.14(0.52 to 2.54)
Education	< 3 years	178(47)	1	159(43)	1	263(37)	1	234(35)	1	230(59)	1	206(51)	1
	≥ 3 years	94(26)	1.07(0.59 to 1.93)	90(25)	0.92(0.46 to 1.82)	140(5)	**0.23(0.09 to 0.56)**	133(5)	**0.23(0.09 to 0.63)**	120(31)	1.01(0.60 to 1.70)	113(30)	1.45(0.82 to 2.57)
	No	47(11)	0.85(0.40 to 1.83)	39(9)	1.39(0.53 to 3.66)	79(15)	1.43(0.74 to 2.77)	65(12)	1.59(0.74 to 3.41)	66(20)	1.26(0.69 to 2.29)	54(17)	1.20(0.60 to 2.41)
Occupation	Working^1^	122(37)	1	114(35)	1	191(18)	1	177(18)	1	175(39)	1	161(35)	1
	Not working^2^	43(17)	1.50(0.71 to 3.18)	36(17)	2.22(0.96 to 5.13)	70(11)	1.79(0.79 to 4.04)	57(10)	1.64(0.71 to 3.82)	56(17)	1.52(0.78 to 2.98)	46(15)	1.43(0.66 to 3.09)
	Retirees^3^	167(31)	**0.52(0.30 to 0.93)**	138(25)	1.02(0.42 to 2.47)	240(30)	1.02(0.42 to 2.47)	198(24)	0.90(0.32 to 2.52)	198(57)	1.41(0.89 to 2.23)	166(48)	1.01(0.42 to 2.41)
Household income	Low/middle	250(61)	1	222(55)	1	377(48)	1	335(44)	1	320(96)	1	286(86)	1
	High	68(22)	1.48(0.79 to 2.77)	66(22)	0.96(0.40 to 2.30)	99(8)	0.60(0.28 to 1.31)	97(8)	1.13(0.40 to 3.16)	88(13)	**0.40(0.21 to 0.77)**	87(12)	**0.36(0.15 to 0.84)**
Household fortune	Small/medium	188(43)	1	171(39)	1	295(39)	1	272(38)	1	258(78)	1	241(71)	1
	Large	120(38)	1.56(0.95 to 2.58)	117(38)	**1.92(1.04 to 3.54)**	165(14)	0.61(0.32 to 1.16)	160(14)	0.59(0.30 to 1.18)	136(29)	0.63(0.38 to 1.04)	132(27)	0.76(0.42 to 1.38)
Smoking	No	252(62)	1	215(56)	1	376(42)	1	323(37)	1	325(79)	1	282(67)	1
	Yes	82(24)	1.27(0.75 to 2.14)	73(21)	1.06(0.59 to 1.92)	126(17)	1.24(0.66 to 2.31)	109(15)	0.99(0.49 to 1.99)	105(35)	1.56(0.99 to 2.46)	91(31)	1.25(0.70 to 2.22)
Alcohol intake per week^4^	≤21	297(73)	1	258(66)	1	449(51)	1	389(46)	1	384(98)	1	335(84)	1
	>21	31(12)	1.94(0.92 to 4.09)	30(11)	1.63(0.70 to 3.79)	45(6)	1.20(0.47 to 3.05)	43(6)	1.43(0.51 to 3.96)	39(14)	1.63(0.81 to 3.30)	38(14)	2.01(0.90 to 4.47)

^1^Employed patients, students

^2^Unemployed patients, patients on disability retirement, patients on personal leave or sick leave not caused by the cancer, housewives

^3^Early retired (age: 60–64) or retired (age: ≥ 65) employee

^4^According to recommendations from the Danish National Board of Health: maximum 21 units per week for men and 14 units per week for women

### Patient delay

Older age predicted shorter *patient delays *(OR 0.30, 95%CI 0.13 to 0.71) than younger age, while patients with a large household fortune experienced longer patient delays (OR 1.92, 95%CI 1.04 to 3.54) than those who were economically less privileged.

### Doctor delay

Well-educated patients experienced shorter *doctor delays *(OR 0.23, 95%CI 0.09 to 0.63) than patients with short education. It is noteworthy that male patients experienced longer doctor delays (OR 2.11, 95%CI 1.11 to 4.02) than female patients.

### System delay

Patients with a high household income experienced shorter *system delays *(OR 0.36, 95%CI 0.15 to 0.84) than economically less privileged patients.

## Discussion

### Summary of main findings

In the case of *patient delay*, retired female patients experienced shorter delays than employed female patients, and female smokers experienced longer delays than female non-smokers. Female patients with a large household fortune experienced shorter *doctor delays *than economically less privileged women, and well-educated male patients experienced shorter doctor delays than less educated men. Male patients had a higher likelihood of experiencing longer doctor delays than women when gender-specific cancers were excluded. Female patients with a large household fortune experienced shorter *system delays *than economically less privileged female patients, while female patients with a high alcohol intake experienced longer system delays than women with an average intake.

### Strengths and limitations of the study

The study encompassed the entire population of newly diagnosed cancer patients in Aarhus County, Denmark. All participants had access to Denmark's tax-financed population-based health care system. We reduced selection bias by using HDR information to identify potential study participants independently of participating GPs and hospital physicians. We were able to confirm patient eligibility by requesting that GPs validate diagnoses and care provided during the inclusion period. If non-participating GPs had relatively more patients with long delays than those who participated, we may have underestimated the number of patients with long delays. However, as only a few of the socioeconomic characteristics were clearly associated with delay, this potential bias may be of limited importance. Furthermore, the high GP response rate (83%) reduced the potential for selection bias.

The selection bias inherent in the 53% patient response rate may have weakened the study. Many patients eligible for study inclusion were old or seriously ill from their cancer or other comorbidities and were not able to comply with our request of filling in the relatively large questionnaire. In addition, some of the non-participating patients may have been socioeconomically disadvantaged and unprepared to complete questionnaires. This may have led to an underestimation of the association between low socioeconomic status and delay.

Minimisation of recall bias is another key prerequisite for the validity of our findings. To this end, we encouraged the GPs to consult their electronic patient files when completing the patient-specific questionnaires. Nearly 100% of the Danish GPs have electronic patient files [23]. To minimise patient recall bias, the patients received the questionnaire as soon as they were identified in the HDR. We are aware that it may be complicated for GPs to accurately define and recall each type of delay, and especially to define "the date of first symptom". We also obtained delay information from patients and compared the GP-reported with the patient-reported delay, and no major discrepancies were found in any of the types of delay (data available on request).

In conclusion, there are few pros and many cons when adopting a questionnaire approach for a study like this. There is a lack of registries on delay information. If such registers had been available, their data would have minimised some of the problems in this study. Until such register information becomes available, the questionnaire approach is, however, supposed to be the best solution.

Gender-specific cancers were excluded from our analyses because treatment paths for women with breast cancer and men with prostate cancer are characterised by short delays for women and long delays for men. As no nationwide screening programme exists for breast or prostate cancer in Denmark, most women with breast cancer have a palpable tumour at the time of first GP consultation and go through a standardised diagnostic investigation. In contrast, men with prostate cancer often have subtle symptoms, and diagnostic strategies are less clearly defined.

Data were analysed using a logistic regression model to estimate the likelihood of long delay. This model was chosen to be able to contrast long with short delay.

Despite the fact that all analyses were based on a solid hypothesis, we cannot exclude that some of our statistically significant findings are caused by multi-significance. Future studies should address this issue.

We pooled all the different cancer diagnoses when analysing the data as one of the main ideas of this study was the adoption of a general practice approach to symptoms, viz. that the patient attends the GP with a symptom that may be related to cancer in general and not to a specific cancer diagnosis, and that the patient seeks help to interpret this symptom. The fear of a serious disease such as cancer, but not of a specific cancer type, is the key element in the patient's help-seeking behaviour [24], and this behaviour is not solely guided by his or her awareness of a possible specific cancer type, but more by personal symptom interpretation. Research into patient delay among breast cancer patients suggests that the patient's initial symptom interpretation, i.e. the stage where the patient determines whether medical attention is required or not, accounts for most of the delay variation [25]. In addition, GPs act on symptoms, too, although their interpretations also encompass a judgment about the "alarmingness" of the symptoms. Finally, the logistics and the capacity in the part of the secondary health care system that primarily performs the diagnostic examinations are not considered to be diagnosis-specific. Given these assumptions, we therefore designed and analysed the study with all cancer diagnoses pooled, well aware that the pooling of delay information for all cancer types may have blurred possible diagnosis-specific associations between delay and patient characteristics.

The population-based approach and the homogeneous structure of general practices in Denmark [18] make the results for Aarhus County generalisable to the rest of Denmark. It is also probable that delay, especially patient delay, is comparable in other countries with a similar health care culture, organisation of medicine and medical capacity as Denmark.

### Comparison with existing studies

Men are generally thought to experience longer delays than women [7,14]. However, Neal and Allgar [9] found longer doctor and system delays among women than among men. Our study revealed that male patients experienced longer doctor delays than female patients when gender-specific cancers were excluded; otherwise no gender differences were found. The gender differences found in other studies may be due to differences in study population, culture and health care system organisation.

Previous studies have found a wide spectrum of associations between delay and patients' socioeconomic characteristics for diagnosis-specific cancers, ranging from no significant predictors to multiple associations. Most studies have focused on characteristics associated with patient delay or doctor delay [7,9,10,15,16]. Few studies have explored socioeconomic characteristics associated with system delay [9], and no clear trend may be discerned from the literature.

### Implications of the study

We found that male patients experienced longer doctor delays than female patients. A possible explanation is that male patients may disclaim or downplay the importance of their symptoms or cancer suspicions when they consult the doctor [12,26]. Another possibility is that the GPs may delay referral because they consider male patients to be overly worried.

Female cancer patients with a large household fortune experienced shorter doctor and system delays than economically less privileged patients. Likewise, well-educated male patients experienced shorter doctor delays than men with short education. Large household fortune and education are probably proxies for more resources and a better ability to describe symptoms, which speeds up referral to further examination or progression within the investigation programme. Another explanation could be that the GPs or hospital physicians relate better to wealthy, well-educated patients and intentionally or unintentionally offer these patients a more rapid diagnostic investigation. Paradoxically, patients in lower socioeconomic groups have high attendance rates to GPs in a gatekeeper system like that in Denmark [27,28]. This is reflected in short patient delays for this group of patients.

Excessive consumption of alcohol and tobacco has been characterised as a self-destructive behaviour, and may correlate with delay in seeking help [29]. Our study provided some confirmation of this hypothesis among female patients, as smoking predicted long patient delays and an excessive alcohol intake predicted long system delays.

The universal access to health care in Denmark might theoretically imply that all patients in the cohort had the same delays. The findings of some differences may indicate that personal differences and differences in symptoms play a role. We found evidence of socioeconomic predictors of delay that allow us to hypothesize the existence of social inequalities in the distribution of delay, but, in general, only few socioeconomic predictors of delay were found. As only small socioeconomic inequalities exist in Denmark [30], our findings concerning socioeconomic characteristics and delay in cancer care do not have major implications for health care provision in this setting.

### Unanswered questions and future research

Future research should focus on symptoms and patient, GP and system characteristics other than socioeconomic factors to clarify predictors of delay. The psychosocial status of the patient, patient comorbidity, GP characteristics and special symptom patterns should be explored.

## Conclusion

Male patients had a higher likelihood of long doctor delays than women when gender-specific cancers were excluded, but apart from this, gender did not predict delay. Female patients with a large household fortune experienced shorter doctor and system delays and well-educated male patients experienced shorter doctor delays than the less privileged patients. We therefore suggest the existence of social inequalities in the distribution of delay.

## Competing interests

The author(s) declare that they have no competing interests.

## Authors' contributions

FO and JS had the idea for the project and acts as guarantors. RPH, JS, HTS and FO designed the project. RPH carried out the study and collected the data. Data processing was by RPH and IS. RPH wrote the first draft of the paper; all authors read and approved the final manuscript.

## Pre-publication history

The pre-publication history for this paper can be accessed here:


